# Advancing interoperability of data exchange in Europe: Insights from Estonia’s experience for the common European data spaces

**DOI:** 10.1016/j.dib.2025.112361

**Published:** 2025-12-07

**Authors:** Anniki Puura, Ralf-Martin Soe, Sara Thabit

**Affiliations:** a FinEst Centre for Smart Cities, Tallinn University of Technology, Ehitajate tee 5, 12616 Tallinn, Estonia; b Academy of Architecture and Urban Studies, Tallinn University of Technology, Ehitajate tee 5, 19086, Tallinn, Estonia; c European Commission Joint Research Centre, Via Enrico Fermi 2749, 21027 Ispra, Italy

**Keywords:** Data space, Interoperability, Data-sharing, Data governance, Smart city, Estonia, X-Road

## Abstract

Interoperability is one of the most critical enablers for the success of the Common European Data Spaces (CEDS). The entry into force of the Interoperable Europe Act in April 2024 marked a pivotal moment in formalising a legal framework to support this vision. Among the emerging data spaces, the European Data Space for Smart Communities (DS4SSCC) has attracted particular attention for its ambition to act as a “connector data space,“ linking stakeholders, platforms, and technologies across local, regional, and national levels, as well as domains, with a focus on community-level challenges and needs.

This paper offers a novel contribution by bridging real-world national and cross-border interoperability practices – particularly Estonia’s operational experience with X-Road – with the strategic development of European-level data space initiatives. Unlike prior studies that focus predominantly on technical frameworks or high-level policy design, this paper provides grounded, practical insights from Estonia’s long-standing implementation and institutionalisation of data exchange interoperability, translating them into actionable recommendations for DS4SSCC and other CEDS.

We argue that the primary obstacles to data exchange interoperability are typically associated with organisational, political, and legal aspects, rather than purely technological ones. Accordingly, effective data governance, stakeholder coordination, and regulatory alignment are essential. To contextualise Estonia’s experience, the paper incorporates a comparative analysis of other key European data space initiatives. This broader perspective situates Estonia’s X-Road model within a diverse ecosystem of approaches, enriching the relevance and applicability of the findings to DS4SSCC and other CEDS. We provide recommendations across three pillars: (1) clear incentives and legal alignment; (2) sustainable organisational structures; and (3) demonstrable value for stakeholders. These aim to guide the DS4SSCC and other CEDS toward becoming scalable, trusted, and inclusive parts of the European data ecosystem.

## 1 Introduction

EU Member States are modernising their public administrations by adopting new digital services. However, such separate initiatives have resulted in the creation of isolated systems, posing challenges to the establishment of the EU’s Digital Single Market [1]. Several EU initiatives have addressed this issue over the past decades – such as the 2017 European Interoperability Framework (EIF) [2] and the ISA Programme [3], among others – with a key milestone in April 2024 marked by the entry into force of the Interoperable Europe Act (2024/903) [4]. According to the EIF, interoperability is defined as *“the ability of organisations to interact towards mutually beneficial goals, involving the sharing of information and knowledge between these organisations, through the business process they support, by means of the exchange of data between their ICT systems.”* However, as the EIF also highlights, effective communication between public administrations requires more than just technical solutions. It also demands agreements, aligned standards, a supportive legal framework and long-term cooperation among organisations. This includes governance structures that enable public administrations at all levels and across sectors, as well as private stakeholders, to work together. Interoperability, therefore, must cover technical, legal, organisational, and semantic aspects [2].

Within this broader framework, “data exchange interoperability” refers specifically to the ability of different ICT systems and organisations – across sectors and borders – to securely, reliably, and meaningfully share and process data in real time or near real time. It encompasses not only technical compatibility (e.g., APIs, data models), but also the institutional and legal mechanisms that enable data to be exchanged in ways that are understandable, actionable, and compliant with applicable regulations. In essence, it ensures that data can move seamlessly between systems and stakeholders to support integrated service delivery, informed decision-making, and innovation.

The EU has undertaken various actions to promote interoperability. Alongside initiatives such as the Single Digital Gateway Regulation [5] and Digital Identity frameworks [6], the EU actively supports the development of Common European Data Spaces (CEDS) through funding and policy measures. These efforts aim to facilitate trusted and secure data sharing across sectors while respecting EU values [7]. Key initiatives supporting the development of EU data spaces include the Data Space Support Centre (DSSC) [8] and Smart Open-source Middleware (Simpl) [9]. Additionally, the European Data Innovation Board (EDIB), established under the Data Governance Act, develops guidelines and frameworks for data governance [10]. According to the DSSC, a data space is *“an interoperable framework based on common governance principles, standards, practices, and enabling services that allow trusted data transactions between participants”* [11]. The European Commission aims to support the establishment of reference architectures, building blocks, interoperability specifications, and data models. Advisory services to guide stakeholders in these efforts are provided by the DSSC [8].

Currently, 14 Common European Data Spaces are under development, each targeting a strategic sector or domain [7]. These include both vertical data spaces – focused on specific domains such as health, mobility, and energy – and horizontal data spaces, which cut across multiple domains, notably the Green Deal Data Space and the European Data Space for Smart Communities (DS4SSCC). Like other evolving data spaces, such as those represented in the IDSA Data Spaces Radar [12], their development is shaped by stakeholders, with the aim of creating features tailored to the unique needs of each domain or cross-domain challenge. All CEDS are expected to adhere to a set of core principles, including: (1) open and inclusive participation for organisations and individuals; (2) privacy-preserving, secure infrastructures for data sharing and processing; (3) transparent, equitable, and trustworthy data governance mechanisms; (4) compliance with EU rules, particularly those protecting personal data and promoting competition; and (5) empowerment of data holders to share data voluntarily, either freely or for compensation [7].

Derived from this, DS4SSCC as an EU data space focused on smart communities faces unique challenges, particularly in addressing the complex governance landscape of smart communities and all the stakeholders involved, as well as context-specific technological solutions already in place at different government levels. In this context, we use the term “smart communities” as outlined in the European Interoperability Framework for Smart Cities and Communities (EIF4SCC). This refers to geographically defined communities within the EU – ranging from capital cities to small villages and surrounding areas – that possess legal status, a legal representative, and self-governance as recognised by their respective Member State. A “smart community” is defined as a sustainable and inclusive community that uses digitally enabled services to improve the quality of life for its residents, businesses, visitors, organisations, and local administrations [13].

The DS4SSCC holds significant potential to become a vital connecting link between the various layers and domains that are crucial for enabling data cooperation to address major challenges faced by communities – such as extreme weather events and the need for sustainable mobility systems. In this context, we conceptualise the DS4SSCC as a “connector data space”: a type of data space that bridges sectors, governance levels, and national borders by enabling interoperability and trusted data exchange across domains, aligning technical, legal, and organisational elements to support cross-cutting cooperation and innovation. At the same time, it faces unique challenges – particularly in navigating the fragmented governance landscape of smart communities and the diversity of context-specific technological solutions already deployed at different levels of government.

To illustrate more how such a “connector data space” can be effectively implemented, this paper focuses on Estonia’s long-standing experience with X-Road – a decentralised, open-source data exchange layer – as a mature, operational example of cross-sectoral and cross-border interoperability. Although the X-Road infrastructure is not officially defined as a data space, it shares several conceptual similarities and offers important lessons for the development of CEDS. While the X-Road protocol provides the technical foundation for secure data exchange, it is complemented by a broader framework of agreements, guidelines, and legal provisions. These elements collectively create the institutional and legal context that enables and encourages public sector organisations to collaborate through data sharing [14].

X-Road has also been federated for cross-border use, notably with Finland [15], allowing the secure exchange of data between national systems – for example, in areas such as population registry queries, health insurance verification, and business registration checks. Estonia’s experience serves as a case study to get actionable insights for scaling DS4SSCC, while also being situated within a comparative analysis of other major European data space initiatives. These examples are used to highlight alternative approaches to governance, incentives, stakeholder engagement, and institutional arrangements, placing Estonia’s X-Road experience within a broader European framework. These examples demonstrate that the primary barriers to interoperability are not technological but institutional, legal and organisational. Accordingly, we argue that aligning incentives, clarifying governance and ensuring demonstrable stakeholder value are essential for success. Drawing on these lessons, this paper presents a roadmap that aims to support the development of DS4SSCC as a scalable, trusted and inclusive part of the European data ecosystem.

The novelty of this study lies in its practical, experience-based analysis of how Estonia’s mature X-Road infrastructure can inform the governance and implementation of the DS4SSCC and other Common European Data Spaces (CEDS). Unlike previous work focused on high-level policy or sectoral pilots, it provides actionable insights grounded in long-term national and cross-border practice to support scalable, community-centred data spaces. While not suggesting that the DS4SSCC must rely on national infrastructure, the paper shows that in smaller countries like Estonia, such an approach has enabled effective data exchange across communities, reduced fragmentation, and supported the inclusion of less-resourced communities in digital governance and service delivery.

## 2 Background

### 2.1 The European data space for smart communities (DS4SSCC)

The European Data Space for Smart Communities (DS4SSCC) is a foundational initiative within the EU data spaces ecosystem, intended to act as a “connector data space.” Aligned with the goals of the European Data Strategy, it plays a crucial role in supporting digital transformation at the local level while promoting interoperability across sectors [16]. As articulated in the European Interoperability Framework for Smart Cities and Communities (EIF4SCC) [13], DS4SSCC is central to achieving the Green Deal objectives by enabling smart city ecosystems to deliver more efficient and integrated services. Interoperability in this context refers to the ability of organisations and individuals to securely and efficiently exchange data, information, and knowledge – essential for cohesive urban planning, mobility, energy and climate adaptation strategies [13].

Local authorities, due to their position as service providers and regulatory agents, are pivotal in this process. Their engagement is supported through tools such as the Data Cooperation Canvas [17] and Minimal Interoperability Mechanisms (MIMs) [18], which are central components of the DS4SSCC blueprint [19]. The first version of the blueprint has delivered: (1) a data governance framework, (2) prioritised datasets, (3) an architectural implementation guide, and (4) a roadmap to maturity – all of which are being further developed as part of ongoing deployment activities. The deployment phase (DS4SSCC-DEP project) builds on it to federate cross-sectoral smart community data spaces using secure, modular middleware components. While pilot projects have begun to test the blueprint’s practical feasibility [16] that feed into the updated version of the blueprint, an unresolved challenge is also the definition of a future-proof organisational model. Because DS4SSCC is inherently horizontal – with a potential to integrate vertical (domain-specific) CEDS through localised applications – it requires an even more comprehensive governance model to connect their stakeholders, sectors and jurisdictions.

### 2.2 The data exchange interoperability in Europe

Achieving interoperability in data exchange across Europe entails navigating a landscape marked by heterogeneous technological infrastructures, complex legal requirements, diverse organisational cultures, and varying business incentives and models. In response, a range of initiatives – both sector-specific and cross-sectoral – have emerged to foster secure, trustworthy, and effective data sharing. These initiatives collectively contribute to the foundations of the Common European Data Spaces (CEDS) and offer essential insights for shaping the European Data Space for Smart Communities (DS4SSCC).

Among the most prominent is Gaia-X initiative established to ensure data sovereignty, cross-sectoral trust, and standardisation [20]. Although Gaia-X introduces critical reference models, its progress has been tackled by implementation challenges and stakeholder coordination issues. Nevertheless, it remains a central actor in shaping EU-wide data governance [21]. Sector-specific initiatives such as Catena-X (automotive) [22] and the European Health Data Space (EHDS) [23] illustrate how domain focus, aligned incentives, and clearly defined governance structures can accelerate adoption and impact. Similarly, MyData Global, originating from the MyData movement and now operating globally, promotes a human-centric approach to personal data governance [24]. For municipalities and public administrations with limited resources, the Simpl middleware – developed under the Digital Europe Programme – is designed to support interoperability between emerging European data spaces while enabling secure data exchange without the need for heavy infrastructure investment [9]. The Data Spaces Support Centre (DSSC) [8], established by the European Commission, plays a coordinating role across these initiatives. It offers technical blueprints, legal guidance, governance models and interoperability frameworks essential to both sectoral and cross-sectoral data spaces. DSSC also fosters a pan-European community of practice, promoting collaboration through working groups, shared documentation, technical dialogues and cross-sectoral workshops. This community-building effort is key to mitigating fragmentation and enhancing coherence across Europe’s digital ecosystem [25].

From a regulatory perspective, the eIDAS Regulation (Electronic Identification, Authentication and Trust Services) provides the legal framework for cross-border digital trust [26]. It ensures mutual recognition of electronic identities and trust services – such as digital signatures and authentication – that are critical for legally compliant and secure data sharing across EU Member States. Complementing these legal foundations is the International Data Spaces Association (IDSA), whose Reference Architecture Model (IDS-RAM) outlines principles such as data sovereignty, usage control and governance by design [27]. These principles are increasingly embedded in Gaia-X and Catena-X, and they are also relevant to DS4SSCC’s technical and governance alignment with EU-wide infrastructures. Collectively, these initiatives and frameworks are key examples addressing the four layers of interoperability – technical, semantic, organisational and legal [2]. Despite the progress made, no single initiative has yet achieved full interoperability across all four layers. Thus, DS4SSCC is well positioned to act as an integrator, leveraging existing frameworks while adapting them to the specific requirements of local governments and communities.

Recent academic work highlights the need for structured, interoperable infrastructures. A comparative study by McBride et al. [28] across 20 countries underscores that data exchange platforms are foundational to digital governance, yet often under-theorised and inconsistently implemented. The study emphasises the importance of legal clarity, modular design, and transparent governance – all essential attributes for DS4SSCC. According to Mergel et al. [29], increased interoperability can be taken as one key objective alongside transparency and citizen satisfaction to improve public sector delivery. Interoperability plays a critical role in digital government and goes beyond computer interoperability (Level 1) which solves technical and semantic issues. More mature models of interoperability include aligned processes (Level 2), shared knowledge (Level 3), shared benefits (Level 4) and ultimately, aligned goals (Level 5).

For DS4SSCC, the goal is not only to enable secure and efficient data exchange but also to foster long-term institutional collaboration and shared value creation at the local level. One of the most instructive and operationalised models of interoperability is Estonia’s X-Road, exemplified in the next chapter. Originally developed in Estonia, it has since been adopted in countries such as Finland, Iceland, Namibia, and Palestine, among others. A comparative study of five countries utilising X-Road, published in 2020, found that Estonia led with 618 services, followed by Finland (190), the Faroe Islands (37), Palestine (30), and El Salvador (0) [30]. X-Road’s success lies in its open-source foundation, legal integration, and robust governance, making it one of the most mature and replicable data exchange infrastructures worldwide. Its real-world application provides critical lessons for DS4SSCC on how to embed interoperability at both technical and institutional levels with focus on community-based needs.

### 2.3 Governing interoperability of data exchange: insights from Estonia

Estonia’s experience with national and international data exchange provides an exemplary model of how digital governance can be effectively implemented when legal, technical, and organisational frameworks are strategically aligned. Although Estonia’s context is unique – particularly due to its lack of legacy systems following independence in 1991 – it offers practical insights for the development of interoperable data spaces across Europe. The country’s approach to data exchange, anchored in the X-Road data exchange layer, demonstrates that successful interoperability depends not solely on technology but on a combination of institutional commitment, regulatory innovation, and broad stakeholder involvement.

#### 2.3.1 National-Level interoperability: The evolution and institutionalisation of X-Road

Estonia’s path toward digital transformation began with a clear political vision and agile policy action. The Principles of Estonian Information Policy (first drafted in 1994, formally adopted in 1998) and the Tiger Leap programme (launched in 1996) laid the groundwork by prioritising digital education and infrastructure. With minimal legacy systems to constrain progress, Estonia implemented X-Road *(X-tee)* – a secure, distributed data exchange layer that has become the backbone of its digital state. Today, 99 % of Estonian public services are online, positioning the country as a global leader in digital governance [31].

**Fig. 1:**
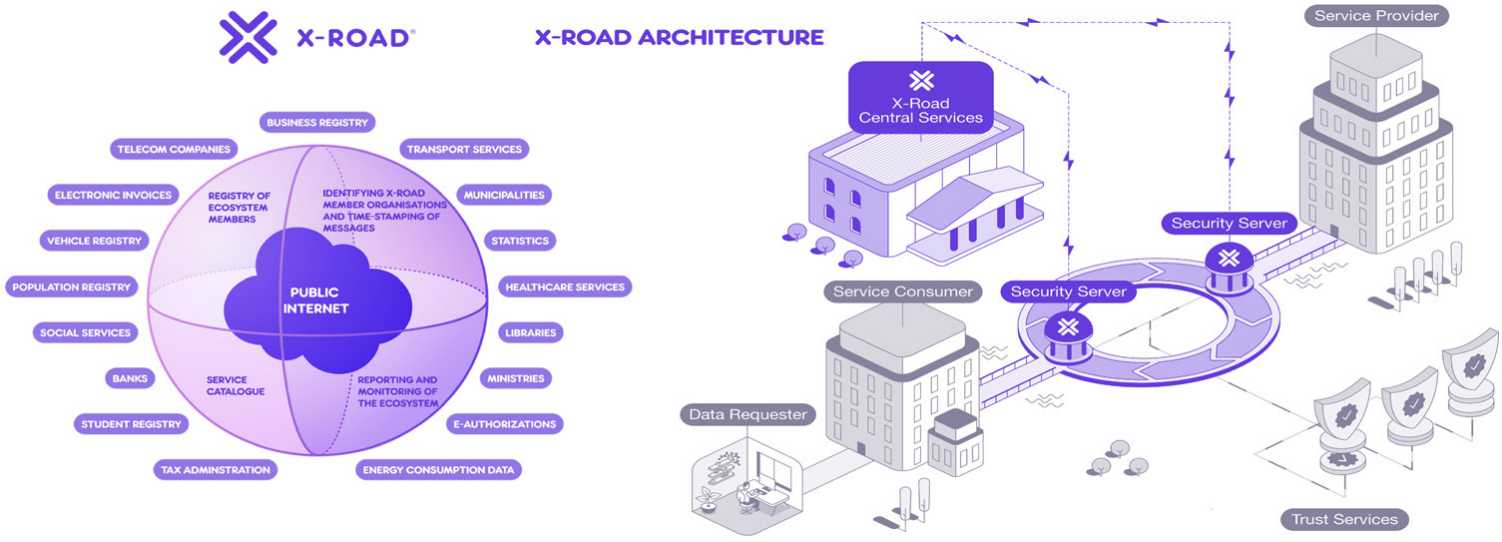
X-Road Ecosystem and Architecture. Source: Official schemes of NIIS [32,33].

As shown in the official NIIS schemes (see Fig. 1), Estonia’s X-Road ecosystem connects public authorities, municipalities, and private organisations via a shared, secure infrastructure. Each participant links through a local Security Server, while the national X-Road Operator manages central services, membership, and trust policies. Certification and time-stamping authorities ensure that data exchanges remain authentic and tamper-proof. Fig. 1 further illustrates how decentralised Security Servers – coordinated by central services – enable authenticated, logged, and traceable data flows across the ecosystem.

X-Road enables interoperability among public-sector databases. A notable example is the national population registry, which is fully integrated with all government institutions, enabling real-time data access and efficient service delivery across the public sector. As a result, municipalities do not maintain their own population registries. Instead, a single live database of all residents in Estonia is used, and every municipality integrates its services with this central registry (e.g., for the registration of new or departing residents).

As Soe [34] notes, the success of X-Road lies not in its technical uniqueness (similar enterprise-service bus platforms exist) but rather due to its successful implementation, both organisationally and legally. The framework follows a rule-based approach, with predefined rules governing who can access specific data and how inquiries are processed [34]. Over the past few decades, X-Road has evolved into an institution, exemplifying North’s [35] concept of the “rules of the game” by establishing clear operational norms for interoperability.

Key legal frameworks have facilitated the wide adoption of X-Road [36]. First, e-Identification was made mandatory in Estonia in the early 2000s, laying the foundation for a digital signing and logging scheme. Second, the Public Information Act, adopted in 2001 [37], established the obligation for all public sector entities to use X-Road to exchange information within state information systems. Additionally, the regulation stipulated that X-Road must be open to private sector users, allowing their participation on a voluntary basis [38]. As Robles et al. [39] describe: *“X-Road serves as a data exchange bus between many databases that implements a set of common features to support and facilitate data exchange. All data exchange is secure, as all outgoing data from X-Road is digitally signed and encrypted, and all incoming data is authenticated and logged. The transversal nature of X-Road makes it possible to not only offer services from Governments, but invites as well participants from the private sector.”* Today, *X-tee* (the official name for Estonia’s X-Road) connects both private and public sector organisations and has made >3000 e-services available [40].

These aspects illustrate Estonia’s legal and technical framework as a key enabler for scaling up interoperability across multiple sectors and stakeholders, offering significant benefits for urban infrastructure and operations. According to Soe [34], the next step for municipalities using X-Road could involve integrating various sensor data by implementing an open and interoperable platform for connected sensors or devices. In this context, beyond citizen-based databases, there could be interconnected registries – both public and private – for “things” such as utility meters (gas, electricity, water), vehicles (cars, buses, trains, etc.), home appliances, heating and lighting systems, waste management systems and weather forecast data. However, the involvement of private companies remains limited, with only a few hundred participating out of all private entities in Estonia, creating a significant barrier to nationwide interoperability. The challenges identified for the voluntary involvement of the private sector in the X-Road infrastructure relate to (1) complexity and bureaucratic issues, including increasing and stricter requirements for security, software, and hardware; (2) additional costs, such as the payment of fees for each query; and (3) little awareness and understanding of the system [41].

Estonia’s legal and technical frameworks could therefore enable future scaling to smart city infrastructure [34]. Cities like Tallinn are already leveraging secure data systems in smart city initiatives [42]. However, further integration – particularly involving the private sector and smaller municipalities – requires sustained institutional support and adaptable governance models. While initiatives like MaaS X-Road aim to integrate mobility services [43], studies indicate that smaller municipalities often lack the financial resources and technical expertise necessary for meaningful participation [44]. This underscores the need for inclusive, multi-level frameworks that align with the principles of the Common European Data Spaces (CEDS), and especially of those the European Data Space for Smart Communities to also address non-technical dimensions of data exchange and the specific needs of diverse communities.

#### 2.3.2 International-Level interoperability: Federated models and cross-border challenges

Additionally, cross-border initiatives have also been undertaken with the aim of expanding this interoperability framework to other countries. For example, in 2014, a decision was made to deploy X-Road infrastructure in Finland as well [45]. Since Finland had already implemented its own data exchange layer, inspired by X-Road, both countries agreed in 2014 to develop a federated solution for interoperability [34]. This process has been led by a joint organisation, the Nordic Institute for Interoperability Solutions (NIIS) [46], which was created with the mission of developing federated e-governance solutions connecting Estonia’s X-Road technology with other interested countries, such as Finland’s *Palveluväylä*. Iceland joined NIIS as a partner in 2019 and became a full member in 2021. Current NIIS partners also include Ukraine, the Faroe Islands, and the Government of Åland [46]. As a standalone solution, X-Road has expanded beyond the Nordic region, now implemented in over 60 countries with 542 million global users. NIIS promotes collaboration, experience sharing, and innovation. With over 4300 members from 162 countries, the X-Road Community enhances the platform through idea exchange, code contributions, and improvements. X-Road’s growth is supported by open-source contributions and strategic partnerships, ensuring continuous development [47].

**Fig. 2:**
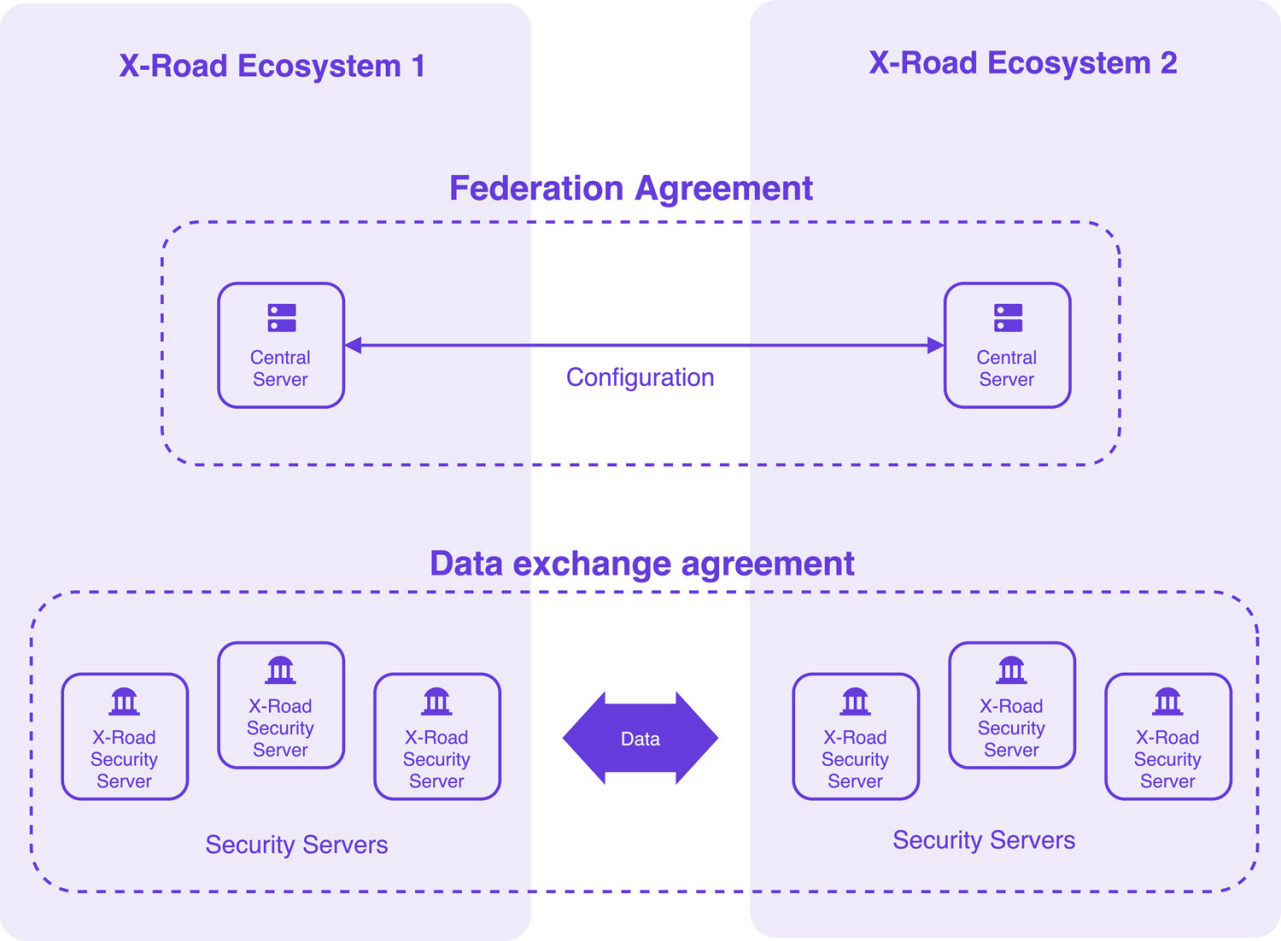
Cross-ecosystem federation architecture. Source: Official scheme of NIIS [48].

Technically, the X-Road software scales to support ecosystems of any size, making it adaptable for many use cases. Two X-Road networks can form a federation to enable secure data sharing. The official NIIS scheme (see Fig. 2) shows how such a federation operates between two ecosystems. To make a federation functional in practice requires more than technical configuration: you also need federation agreements to establish mutual system linkage and trust, data-exchange agreements to specify exactly what data is shared, when, and by whom, and governance frameworks to uphold accountability and ensure compliance across both networks.

It therefore also enables cross-border data exchange through federated ecosystems, where two X-Road networks establish a federation to facilitate secure data sharing for national and international purposes [49]. Experience from Estonia and Finland shows that mutual collaboration and legal requirements have been critical in allowing cross-border data exchange [45]. The fact that X-Road was not supported by EU-level legislation required specific agreements between interested parties to facilitate data exchange. For example, data exchange in areas such as business registers, population registers and tax administrations started with joint national declarations, followed by technological solutions and institutional agreements between the relevant registers [45].

Therefore, the implementation of federated data exchange layers across countries has not only been a technological challenge, but rather its main barriers have been legal and organisational. Effective governance structures, including multi-party agreements on data usage rules, remain essential for scaling interoperability beyond national contexts. To align with the broader Common European Data Spaces (CEDS) vision, NIIS is currently working to integrate X-Road with the Gaia-X trust framework through the upcoming X-Road 8 “Spaceship” release. While the current broader X-Road framework ensures technical compatibility, full alignment with Gaia-X will require meeting additional governance and compliance requirements [50]. As highlighted by NIIS, the ongoing efforts to enable interoperability between X-Road and other data exchange ecosystems – including emerging European data spaces – are not only vital for advancing cross-border data exchange, but also for enhancing and future-proofing X-Road itself.

To conclude, Estonia’s experience shows that secure and interoperable data exchange depends on the alignment of legal frameworks, technical infrastructure, and institutional coordination. While early success was aided by favourable starting conditions, Estonia’s sustained investment in digital governance, open-source data exchange layer, and collaborative structures offers valuable lessons. Replicating this model across Europe requires adapting to varied legal, institutional, and local contexts. Estonia’s experience highlights the importance of aligning local initiatives – particularly those already implemented at national or cross-border levels – with overarching EU frameworks. For DS4SSCC, this means combining proven practices with inclusive governance and support for smaller municipalities and private actors.

Federated models such as those developed through NIIS offer promising templates for cross-border interoperability, but their success relies on shared legal frameworks, aligned incentives, and sustained political will. As Europe moves toward integrated data ecosystems, Estonia’s journey provides a strong reference point for building scalable, trustworthy, and citizen-centred data spaces. Drawing from these insights presented, several key recommendations emerge for the development and operationalisation of the European Data Space for Smart Communities (DS4SSCC), as well as for Common European Data Spaces (CEDS) more broadly, as they also tend to involve multiple levels from local to EU.

## 3 Recommendations

The establishment and widespread deployment of new technological data exchange solutions involve complex interactions with various governance, legal and business processes and arrangements. These interactions become even more intricate when considering initiatives such as the European Common Data Spaces, particularly the European Data Space for Smart Communities (DS4SSCC) aiming to involve a wide range of stakeholders across sectors and EU countries. At the same time, interoperability is both a necessary condition for the implementation of data spaces and a potential driver for the DS4SSCC to be conceived and leveraged as a 'connector data space,' linking various levels – from local to EU – as well as different sectors and Common European Data Spaces (CEDS), with a focus on communities.

Drawing from Estonia’s lessons – and recognising broader challenges such as stakeholder buy-in, uneven local capacities and enforcement complexity – this section presents actionable recommendations grounded in practical experience and complemented by comparative insights from initiatives such as Gaia-X, Catena-X, EHDS, and Simpl. These initiatives collectively demonstrate the importance of tailoring governance and incentive mechanisms to different sectoral, legal, and institutional contexts, which DS4SSCC must navigate. Recommendations are accompanied by potential barriers, mitigation strategies and references to models or architectures (such as IDSA, DSSC, Gaia-X, Catena-X and NIIS) that offer implementation guidance. These steps aim to make DS4SSCC both operational and scalable within the larger ecosystem of CEDS.

**Fig. 3:**
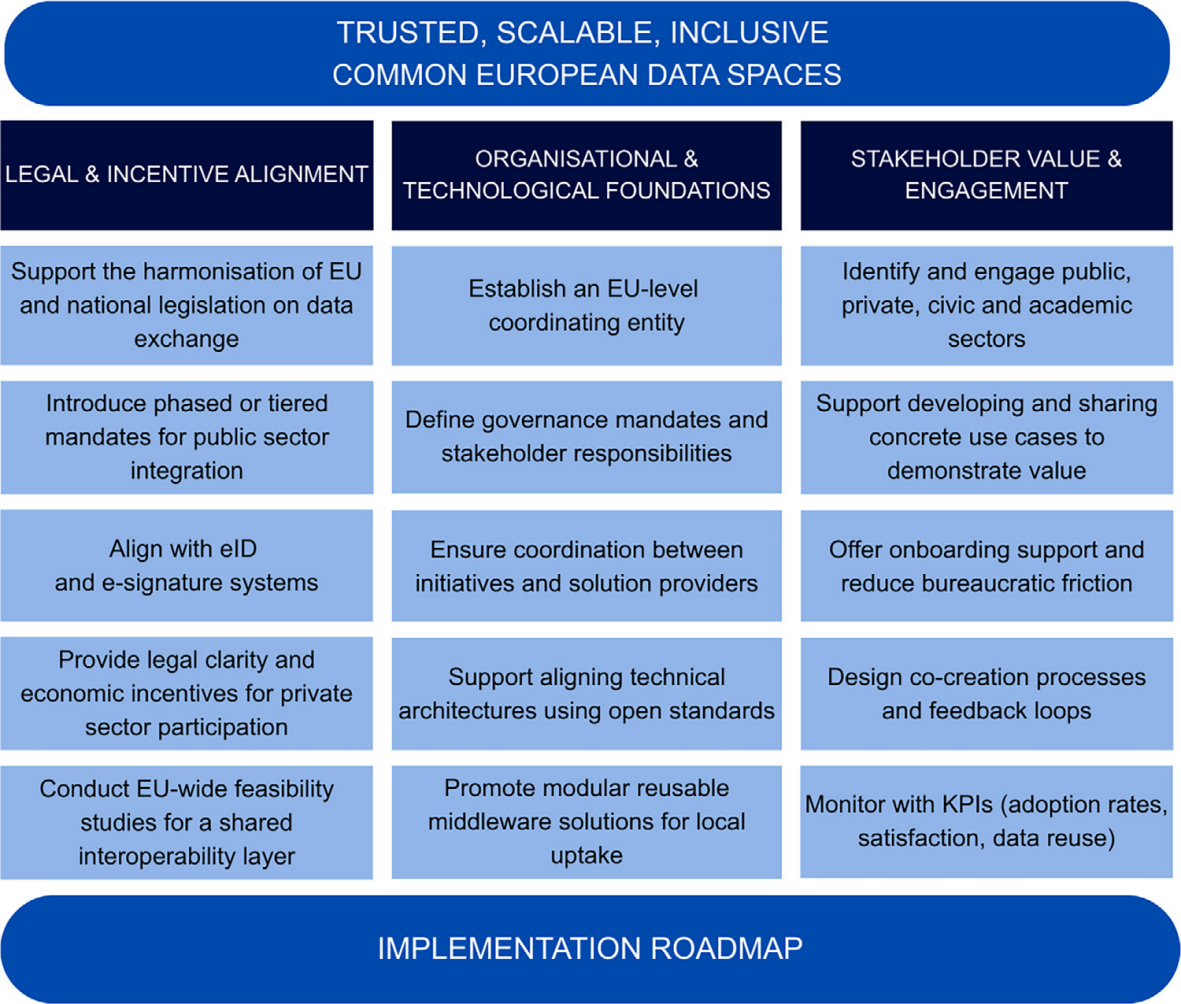
Roadmap for Implementing Common European Data Spaces (CEDS). Source: Authors’ own elaboration.

Based on an analysis of Estonia’s experience as well as relying on other examples of data exchange interoperability in European context, we present three interconnected recommendations to consider and incorporate into the development of the DS4SSCC to fully exploit its potential as a driver for interoperability across smart communities data spaces as well as CEDS: (1) Clear incentives and legal alignment, (2) Organisational arrangements and technological developments, and (3) Value creation for stakeholders (Fig. 3).

### 3.1 Establish clear incentives and legal alignment

One of the main success factors behind the adoption of the interoperability data exchange layer in Estonia was the mandatory implementation of the data exchange layer for public sector information. This enabled the broad adoption of the technological solution across public sector organisations and the establishment of a ready-to-use infrastructure backbone for interoperability. Even though mandatory integration with Estonia’s data exchange layer can be effective for interoperability, the challenge is that it has not been successful in terms of attracting external partners (e.g. smaller businesses) voluntarily to exchange information and that scaling the model outside Estonia requires significant institutional and regulatory alignment, as seen in limited Nordic adoptions.

When considering the DS4SSCC, political will and support at the EU, national, and local levels must be mobilised to actively develop and enforce effective governance and interoperability frameworks, along with the supporting technologies. This aspect is particularly relevant for the design and implementation of regulatory obligations at the EU level, which may be necessary to ensure the harmonised adoption of the technology required for interoperability [51]. However, enforcing mandatory implementation across highly diverse contexts presents a risk of low compliance or pushback. To mitigate this, a tiered or phased enforcement model could be introduced, allowing local adaptation while preserving strategic alignment [52]. Gaia-X and the upcoming European Health Data Space (EHDS) provide instructive contrasts: while Gaia-X has struggled with broad adoption due to unclear incentives and complex federated governance, the EHDS is expected to benefit from strong sectoral mandates and a clear regulatory foundation. These comparisons reinforce that successful interoperability initiatives require both legal certainty and stakeholder buy-in, a balance that DS4SSCC must deliberately cultivate.

At present, the launch of the EU Digital Identity Wallet [53] could represent an initial step toward a digital signing and logging scheme that facilitates citizen-centric interoperability, as demonstrated by the Estonian experience. EU institutions and Member States should initiate a feasibility study and stakeholder consultations to explore the mandatory adoption of a pan-European interoperability layer for public sector information. This exploration should:

• Assess the possibilities and limitations of such a mandate.
• Evaluate the implications of choosing a specific technology solution (e.g., Simpl, which is currently under development as a technical enabler for interoperability rather than as a governing framework [9]).
• Ensure compatibility with existing local and national infrastructures (e.g., X-Road).

Additionally, for the DS4SSCC to drive successful interoperability across data spaces, further considerations should be given to private sector entities and their engagement as well. In this context, there is a need to:

• Design a combined framework of legal and regulatory obligations with incentives for voluntary adoption.
• Simplify administrative processes and reduce operational complexity.
• Introduce financial support mechanisms to offset additional compliance costs.
• Launch awareness campaigns and capacity-building programmes to promote adoption among stakeholders [39].

In parallel, a targeted programme of empirical research and data collection should be initiated to inform the implementation of these actions and provide practical guidance for the way forward. The key questions of this research should specifically aim to:

• Evaluate the feasibility and policy impact of mandating a pan-European data exchange layer for public sector information.
• Analyse the consequences of standardising on a single interoperability technology across the EU, including integration pathways for existing local systems.
• Define a set of incentives that would effectively encourage private-sector participation in a European interoperability framework on a voluntary basis.
• Review current EU regulations, cross-border agreements, and sectoral laws governing data sharing, and determine how existing legal bases could be leveraged to mandate data exchange in critical contexts, such as crisis management.

These efforts should be guided by a multi-level governance model that balances EU-wide harmonisation with flexibility for national and local adaptation. Drawing from DSSC and IDSA’s frameworks, standard implementation models and legal interoperability checklists can be adapted and disseminated to support stakeholder compliance and coherence.

### 3.2 Establish sustainable organisational structures

In addition to binding rules and political support, robust and sustainable organisational structures are essential to the development and implementation of interoperability frameworks and technologies. The Estonian experience illustrates clearly: scaling up interoperability led to the creation of the Nordic Institute for Interoperability Solutions (NIIS), a dedicated institutional entity including organisations from Estonia, Finland and Iceland. NIIS is collectively responsible for providing and maintaining the interoperability framework and the activities of these organisations are supported by a strong global community. By comparing with organisational models like the Gaia-X Association or Catena-X’s governed consortium approach, we observe different degrees of stakeholder coordination and centralisation. DS4SSCC should adopt a hybrid strategy, drawing on both federated, multi-stakeholder governance models (as in Gaia-X and X-Road) and sector-focused structures (as in Catena-X) to balance flexibility with accountability. This enables a continuous understanding of the technology users’ needs and ensures that the technology remains flexible and adaptable. As described, NIIS functions as both a network and cooperation platform, and an executor of IT developments in members' common interests [39].

In other words, the creation of a new organisational entity with members from the involved countries has been crucial to fostering and maintaining a strong connection between the community and technological developments. The X-Road initiative, supported by NIIS, not only delivers an interoperability solution – including the provision of open-source software for the use of public and private stakeholders – but also creates a multi-stakeholder community that involves end users and citizens [39].

For the DS4SSCC, it is essential to identify which organisation(s) could take on the long-term responsibility for managing interoperability in EU data spaces and how they will ensure consistency and continuous development according to the needs of data space users. Academic research confirms that the early and continuous involvement of stakeholders and users in the technology development process increases the quality and legitimacy of the final solution [54].

The DS4SSCC should therefore support the creation of a new EU-level organisational entity dedicated to managing interoperability across European data spaces. This body should be anchored in the existing DS4SSCC network, allowing it to operate across governance levels – from local communities to the EU – and to represent diverse domains such as mobility, energy, and basic public services. Its role would be to connect stakeholder needs with evolving technologies and regulatory developments, ensuring responsiveness, continuity, and coherence. The main functions of such an entity would revolve around the representation of cross-domain needs, accounting for the specific needs of local communities, and providing advice on revising and developing political and regulatory frameworks across Europe, in close coordination with the European Data Innovation Board (EDIB) [10].

To ensure effective operation, specific criteria should be developed to assess performance and alignment with broader CEDS objectives. These could include stakeholder satisfaction, interoperability maturity levels, system reuse rates and alignment with DSSC architectural blueprints. Implementation guidelines, building on Gaia-X governance frameworks and IDSA’s RAM model [27], can provide technical and procedural clarity.

Rather than replacing ongoing technical development initiatives like Simpl, this entity should work in a complementary role, acting as coordinator and execution platform that ensures technology evolves in line with user needs and societal goals. To achieve this vision, several parallel efforts should be supported:

• Developing a clear institutional model grounded in the DS4SSCC network, with defined participation pathways for public authorities, private actors, academia and civil society.
• Defining a governance structure and mandate that allows the entity to represent local and cross-sector needs, advise on political and regulatory updates, co-design and test new interoperability tools and maintain strong stakeholder engagement mechanisms.
• Establishing formal coordination channels with the current technology developer for interoperability in data spaces (i.e., Simpl).
• Launching research and stakeholder consultations to map existing relevant organisations, assess legal, governance and business model implications, explore alignment with the European Data innovation Board (EDIB) and the Data Spaces Support Centre (DSSC), and identify long-term funding and operational requirements.

Through these efforts, the DS4SSCC can contribute to building a durable and inclusive organisational infrastructure that sustains both the technological and community-driven dimensions of interoperability. This will be fundamental for the success and scalability of data spaces across the EU.

### 3.3 Create and demonstrate value for stakeholders

Although successful measures and frameworks have been adopted in Estonia regarding public sector interoperability, the full engagement of private stakeholders remains an ongoing challenge that requires further effort [41]. As companies grow increasingly dependent on information technologies, interoperability has been recognised as a catalyst of innovation [55]. Academic studies, such as the one conducted by Schladofsky et al. [56], identify the diversity of stakeholder business models within interoperable IoT ecosystems, illustrating that “all stakeholders can profit from interoperability.” However, the value of interoperability is often not immediately apparent to many stakeholders, with the current landscape marked by low levels of awareness and limited understanding of the benefits associated with their engagement in interoperable ecosystems [41].

Across various initiatives, value creation strategies differ. For example, Catena-X demonstrates the power of supply chain use cases to drive business participation, while MyData leverages ethical incentives tied to user control. The DS4SSCC should explore a pluralistic value strategy that incorporates both economic and social incentives tailored to the local smart community context.

From the perspective of the DS4SSCC as a potential driver of interoperability, it is necessary to gain a broader understanding of the value perspectives of different stakeholders – public, private sector, academia, and the third sector – and the various approaches to value creation they may have. Achieving this requires adopting co-creation methods to both understand and shape interoperability frameworks [53]. Such participatory approaches help align different and often competing interests typical of multi-sector, multi-actor contexts like smart cities [57], while also building and sustaining trust in the interoperable layer, a crucial factor for its successful uptake [58].

To further strengthen the case for engagement, it is crucial to demonstrate value through clear and practical use cases. These use cases serve a dual role: they help design incentives tailored to different stakeholders, and they increase awareness of tangible benefits and potential business models emerging from the interoperable layer. Since value creation benefits significantly from economies of scale, encouraging broad participation in the data exchange layer is paramount. The more stakeholders engage in the data exchange layer, the more likely new business models can be developed [57].

Accordingly, concrete steps should be taken to lower the barriers for new participants entering the ecosystem, including:

• Exploring and implementing automated orchestration processes that streamline user onboarding and empower stakeholders to adapt quickly and effectively.
• Aligning legal frameworks across member states to reduce complexity and ensure interoperability requirements are clear, harmonised and supportive of innovation [56].
• Simplifying administrative and bureaucratic procedures to remove unnecessary obstacles and facilitate faster, easier access to the interoperable data exchange layer [41].
• Designing and promoting tailored incentives that motivate diverse stakeholders to participate and contribute to the ecosystem.
• Ensuring ongoing stakeholder engagement and feedback loops to continuously adapt and improve entry processes and ecosystem governance.

To assess the success and applicability of these measures, DS4SSCC and other CEDS should introduce key performance indicators (KPIs) such as participation growth rate, number and diversity of interoperable use cases, time-to-integration and stakeholder satisfaction. By aligning these metrics with goals defined by initiatives like Catena-X and the DSSC maturity roadmap, stakeholders can monitor progress and course-correct as needed.

These actions will help to create a more inclusive and accessible data exchange ecosystem, driving widespread adoption and unlocking new value for all participants. By enabling innovative business models and reinforcing trust among stakeholders, this ecosystem will play a pivotal role in advancing the digital transformation of cities and communities throughout Europe.

## 4 Conclusions

Interoperability continues to be both a foundational challenge and a transformative enabler for Common European Data Spaces. As this paper has shown, achieving effective interoperability requires more than technological alignment – it also depends on legally enforceable frameworks, institutional commitment, and coordinated cooperation across diverse stakeholders and governance levels.

The European Data Space for Smart Communities (DS4SSCC) stands out for its ambition to operate as a cross-domain and multilevel “connector data space”. Its success will depend on learning from real-world examples – particularly Estonia’s long-standing experience with the data exchange layer X-Road. However, Estonia’s experience alone does not provide all the answers. By integrating comparative insights from initiatives such as Gaia-X, Catena-X, EHDS, and others, this paper demonstrates how different data spaces confront common challenges in governance, incentives, legal frameworks, and stakeholder participation.

At the same time, the Estonian example also reveals important limitations – especially in engaging the private sector. Complex onboarding procedures, high compliance costs and limited incentives have slowed private participation. If DS4SSCC and other CEDS are to function as truly federated and inclusive data spaces, these challenges must be addressed through simplified governance processes, reduced entry barriers, and clearer value propositions for non-public stakeholders.

Looking ahead, DS4SSCC must be equipped to meet the demands of future data ecosystems. From integrating real-time sensor networks to enabling interoperability across smart infrastructure and IoT platforms, the scope of interoperability must expand beyond current technical and legal dimensions. The rise of AI in public services – such as predictive analytics, urban management or personalised services – will depend on reliable, high-quality data exchange across domains. Without robust interoperability, the benefits of such technologies will remain limited in scale and accessibility.

The broader EU data space landscape should promote cross-learning by encouraging major initiatives – such as Gaia-X, Simpl, and IDSA – to share operational insights. Estonia’s transparent implementation of X-Road offers a strong model. Rather than pursuing isolated pilots, DS4SSCC and related efforts must coordinate strategically to deliver value across local, national, and European levels. By aligning with EU-wide initiatives and embedding comparative perspectives, DS4SSCC can become a key pillar of the CEDS. Upholding principles like legal clarity, institutional resilience, and shared governance will be essential to building a scalable and trusted digital infrastructure – reliable not only for governments, but also for citizens, businesses, and communities.

## CRediT Author Statement

**Anniki Puura:** Conceptualisation, Methodology, Investigation, Writing – review & editing. **Sara Thabit:** Conceptualisation, Methodology, Investigation, Writing – review & editing. **Ralf-Martin Soe:** Conceptualisation, Methodology, Investigation, Writing – review & editing. All authors have read and agreed to the published version of the manuscript.

## Ethics Statement

The authors have read and followed the ethical requirements for publication in Data in Brief and confirm that the current work does not involve human subjects, animal experiments, or any data collected from social media platforms.

## Declaration of Generative AI and AI-Assisted Technologies in the Writing Process

As the authors’ native language is not English, they used ChatGPT to check the language and grammar during the preparation of this work. After using this tool, the authors reviewed and edited the content as needed and take full responsibility for the publication’s content.

## Declaration of Generative AI in Scientific Writing

In this study, generative AI tools were used solely for language correction, enhancing readability, and improving the clarity of the text. The AI was not involved in data analysis, research design, or the formulation of ideas. All intellectual content and scientific contributions remain the responsibility of the authors.

## Data Availability

X-Road Document Library (Reference data). DS4SSCC website (Reference data). NIIS website (Reference data). e-Estonia website (Reference data).

## References

[1] European Commission, A Digital Single Market Strategy for Europe. Communication from the Commission to the European Parliament, the Council, the European Economic and Social Committee and the Committee of the Regions, 2015. https://ec.europa.eu/commission/presscorner/api/files/attachment/8210/DSM_communication.pdf (accessed 09 December 2024).

[2] European Commission, European Interoperability Framework – Implementation Strategy. A digital Single Market Strategy for Europe. Communication from the Commission to the European Parliament, the Council, the European Economic and Social Committee and the Committee of the Regions, 2017. https://eur-lex.europa.eu/resource.html?uri=cellar:2c2f2554-0faf-11e7-8a35-01aa75ed71a1.0017.02/DOC_1&format=PDF (accessed 09 December 2024).

[3] Decision (EU) 2015/2240 establishing the ISA² programme, 25 November 2015. https://ec.europa.eu/isa2/sites/default/files/celex_en.pdf (accessed 09 December 2024).

[4] Regulation (EU) 2024/903 of the European Parliament and of the Council of 13 March 2024 laying down measures for a high level of public sector interoperability across the Union (Interoperable Europe Act). https://eur-lex.europa.eu/eli/reg/2024/903/oj (accessed 09 December 2024).

[5] Regulation (EU) 2018/1724 establishing a single digital gateway to provide information, procedures, assistance, and problem-solving services. http://data.europa.eu/eli/reg/2018/1724/oj (accessed 09 December 2024).

[6] European Commission, European Digital Identity. https://commission.europa.eu/strategy-and-policy/priorities-2019-2024/europe-fit-digital-age/european-digital-identity_en (accessed 09 December 2024).

[7] European Commission, Common European Data Spaces. https://digital-strategy.ec.europa.eu/en/policies/data-spaces (accessed 09 December 2024).

[8] Data Spaces Support Centre. https://dssc.eu/ (accessed 09 December 2024).

[9] European Commission, Simpl: cloud-to-edge federations empowering EU data spaces. https://digital-strategy.ec.europa.eu/en/policies/simpl (accessed 09 December 2024).

[10] Regulation (EU) 2023/2854 of the European Parliament and of the Council of 13 December 2023 on harmonised rules on fair access to and use of data and amending Regulation (EU) 2017/2394 and Directive (EU) 2020/1828 (Data Act). https://eurlex.europa.eu/eli/reg/2023/2854 (accessed 09 December 2024).

[11] Data Spaces Support Centre, introduction – Key concepts of Data spaces. https://dssc.eu/space/bv15e/766061351/Introduction+-+Key+Concepts+of+Data+Spaces (accessed 09 December 2024).

[12] IDSA Data Spaces Radar, https://www.dataspaces-radar.org/radar/ (accessed 09 December 2024).

[13] European Commission: Directorate-General for Communications Networks (2021). https://data.europa.eu/doi/10.2799/816559.

[14] Kivimäki, P.. Is X-Road a data space technology? Nordic Institute for Interoperability Solutions, 2023. https://www.niis.org/blog/2023/6/21/is-x-road-a-data-space-technology (accessed 09 December 2024).

[15] X-road history. https://x-road.global/xroad-history (accessed 09 December 2024).

[16] European data space for smart communities (DS4SSCC), call for pilots manual. https://www.ds4sscc.eu/s/CfP_Call-for-Pilots-Manualdocx_compressed.pdf (accessed 09 December 2024).

[17] R. van der Lans, J. Soetendal, Data cooperation canvas. URL: https://www.datacooperationcanvas.eu/ (accessed 09 December 2024).

[18] OASC, Minimal interoperability mechanisms – MIMs – explained. https://oascities.org/minimal-interoperability-mechanisms/.

[19] European Data Space for Smart Communities (DS4SSCC), DS4SSCC Blueprint. URL: https://www.ds4sscc.eu/Blueprint (accessed 09 December 2024).

[20] Gaia-X: a federated secure data infrastructure. https://gaia-x.eu/about/ (accessed 06 June 2025).

[21] GXFS. Implementation strategies for the Gaia-X Federation Services: project progress and new requirements – A delta study with outlook, October 2023. https://www.gxfs.eu/gxfs-delta-study/.

[22] Catena-X: your automotive network. https://catena-x.net/ (accessed 06 June 2025).

[23] The European Health Data Space (EHDS) https://www.european-health-data-space.com/ (accessed 06 June 2025).

[24] MyData. https://mydata.org/ (accessed 06 June 2025).

[25] DSSC: community of Practice https://dssc.eu/space/DC/27983886/Community+of+Practice (accessed 06 June 2025).

[26] European Commission: eIDAS Regulation. https://digital-strategy.ec.europa.eu/en/policies/eidas-regulation (accessed 06 June 2025).

[27] IDSA – Reference Architecture. https://internationaldataspaces.org/offers/reference-architecture/ (accessed 06 June 2025).

[28] McBride K., Kamalanathan S., Valdma S.M., Toomere T., Freudenthal M. (2022). Proceedings of the 15th International Conference on Theory and Practice of Electronic Governance.

[29] I. Mergel, N. Edelmann, N. Haug. Defining digital transformation: results from expert interviews Government Information Quarterly, 36, Issue 4, October 2019, 101385. 10.1016/j.giq.2019.06.002.

[30] Saputro R., Pappel I., Vainsalu H., Lips S., Draheim D. (2020). 2020 Seventh International Conference on eDemocracy & eGovernment (ICEDEG).

[31] e-Estonia, story. https://e-estonia.com/story/ (accessed 09 December 2024).

[32] NIIS. X-Road technology overview. https://x-road.global/x-road-technology-overview (accessed 17 October 2025)

[33] NIIS. X-road architecture. https://x-road.global/architecture (accessed 17 October 2025)

[34] Soe R.-M. (2018). https://digikogu.taltech.ee/en/item/50b3f649-0691-4263-93c2-4ce6d129b376.

[35] North D.C. (1994). Economic performance through time. Am. Econ. Rev..

[36] Jackson E.B., Dreyling R., Pappel I. (2022). Proceedings of the 14th International Conference on Theory and Practice of Electronic Governance (ICEGOV '21).

[37] Riigikogu, Public Information Act [RT I 2000, 92, 597]. https://www.riigiteataja.ee/en/eli/ee/518012016001/consolide/current (accessed 09 December 2024).

[38] PRC/RIA, Relevant national regulation for the execution of Trust Federation, 28 June 2017. https://www.ria.ee/sites/default/files/documents/2022-11/Trust-Federation-Appendix-2-Regulation.pdf (accessed 09 December 2024).

[39] Robles G., Gamalielsson J., Lundell B., Lindgren I. (2019). Electronic Government. EGOV 2019, Lecture Notes in Computer Science.

[40] e-Estonia, X-Road Interoperability Services. https://e-estonia.com/solutions/x-road-interoperability-services/x-road/ (accessed 09 December 2024).

[41] Paide K., Pappel I., Vainsalu H., Draheim D. (2018). ICEGOV '18: 11th International Conference on Theory and Practice of Electronic Governance.

[42] e-Estonia, What is a smart city? The Estonian vision for new models of smart city. https://e-estonia.com/the-estonian-smart-city/ (accessed 09 December 2024).

[43] Republic of Estonia Transport Administration, integrated route planning and ticket purchasing platform to simplify travelling in Estonia. https://transpordiamet.ee/en/news/integrated-route-planning-and-ticket-purchasing-platform-simplify-travelling-estonia (accessed 09 December 2024).

[44] e-Estonia, How to digitalise local governments? https://e-estonia.com/how-to-digitalise-local-governments/ (accessed 09 December 2024).

[45] Krimmer R., Solvak M., Alishani A., Dedovic S., Schmidt C., Castle S. (2022). https://static1.squarespace.com/static/59ba41ee64b05fd6531f498d/t/62b476a8e7006b54f8488c34/1655994030796/NIIS_Interoperability_Landscape_Report_2022.pdf.

[46] Nordic Institute for Interoperability Solutions (NIIS). https://www.niis.org/ (accessed 09 December 2024).

[47] X-Road, community. https://x-road.global/community (accessed 09 December 2024).

[48] NIIS. X-Road Trust Federation. https://x-road.global/trust-federation (accessed 17 October 2025)

[49] NIIS, X-road implementation models. https://www.niis.org/blog/2020/3/30/x-road-implementation-models (accessed 09 December 2024).

[50] NIIS, NIIS announces proof of concept for revolutionary X-road 8 “Spaceship” https://www.niis.org/blog/2023/12/20/niis-announces-proof-of-concept-for-revolutionary-x-road-8-spaceship- (accessed 17 October 2025).

[51] R. Silvestro, F.J. Schenatto, G.D. Santos, G.A. Oliveira, Acceptance of a mandatory e-government system: the public employee perspective, Int. J. Electron. Gov. Res. 20 (2024) 1–19. 10.4018/IJEGR.356406.

[52] Black J. (2008). Constructing and contesting legitimacy and accountability in polycentric regulatory regimes. Regul. Gov..

[53] European Commission, EU Digital Identity Wallet. https://ec.europa.eu/digital-building-blocks/sites/display/EUDIGITALIDENTITYWALLET/EU+Digital+Identity+Wallet+Home (accessed 09 December 2024).

[54] Flores C.C., Rodriguez Müller A.P., Virkar S., Temple L., Steen T., Crompvoets J. (2022). Towards a Co-creation approach in the European Interoperability Framework, transforming government: people. Process and Policy.

[55] Eloff J.H.P., Eloff M.M., Dlamini M.T., Ngassam E., Ras D., Zelm M. (2013). Enterprise Interoperability: Research and Applications in the Service-oriented Ecosystem: proceedings of the 5th International IFIP Working Conference IWEI 2013.

[56] Schladofsky W., Mitic J., Megner A.P., Simonato C., Gioppo L., Leonardos D., Bröring A., Žarko I.P. (2017). Interoperability and Open-Source Solutions for the Internet of Things. InterOSS-IoT 2016, Lecture Notes in Computer Science.

[57] Thabit S., Mora L. (2024). The collaboration dilemma in smart City projects: time to ask the right questions. Organization.

[58] Reimers K., Guo X. (2024). AMCIS 2024 Proceedings.

